# Robotic assisted Laparoscopic partial Nephrectomy for suspected Renal Cell Carcinoma: Retrospective review of surgical outcomes of 35 Cases

**DOI:** 10.1186/1471-2482-8-16

**Published:** 2008-09-24

**Authors:** Sam B Bhayani, Nitin Das

**Affiliations:** 1 Department of Surgery, Division of Urology, Washington University School of Medicine, Saint Louis, MO, USA

## Abstract

**Background:**

A standard of care for the treatment of small renal masses is partial nephrectomy. The open and laparoscopic approaches have been well described in the literature. Robotic assistance may augment partial nephrectomy by aiding in dissection and renal reconstruction. In this communication we describe the surgical outcomes of 35 patients undergoing robotic partial nephrectomy.

**Methods:**

Patient records and databases were reviewed for 35 consecutive patients undergoing RPN. Clinical, pathological, and radiographic data were obtained. The data was deidentified.

**Results:**

Thirty five patients successfully underwent RPN. An additional 2 patients were converted to other nephron sparing procedures. Mean tumor size was 2.8 cm, and mean OR time was 142 minutes. Mean warm ischemia time was 20 minutes. All margins were negative. There were 4 complications, and no patients required reoperation.

**Conclusion:**

Robotic partial nephrectomy can produce excellent initial results. Further studies should be performed to compare the outcomes to laparoscopic and open operations.

## Background

The small suspicious renal mass may be treated with a variety of modalities. Open radical nephrectomy is the traditional treatment for a renal neoplasm, but open partial nephrectomy has evolved into a standard of care, with the obvious advantage of sparing the kidney.[1] In the 1990s laparoscopic approaches to partial nephrectomy were developed.[2,3] The laparoscopic partial nephrectomy has been performed in centers of excellence with reasonable results.[4] However, the operation has also been thought to be technically advanced secondary to the laparoscopic reconstructive skills necessary to perform the procedure quickly while the kidney is under warm ischemia.

Robotic surgical assistance has been used to perform complex reconstructive procedures in a minimally invasive fashion. Robotic radical prostatectomy has become the prime example in which a complex open procedure may be reproduced with robotic assistance in a minimally invasive fashion.[5] The da Vinci robot (Intuitive Surgical, Sunnyvale, CA, USA) allows ease of intracorporeal dissection and suturing secondary to the wristed and articulating instrumentation. To date, the robotic system has been sparsely reported as an adjunct to laparoscopic partial nephrectomy. [6-11] In this series, we report the outcomes of 35 patients undergoing robotic assisted laparoscopic partial nephrectomy (RPN), which represents one of the largest series.

## Methods

A retrospective review was performed of 35 patients undergoing RPN after institutional board approval and compliance with the Helsinki Declaration. In all cases, a suspicious enhancing renal mass or complex enhancing renal cyst was present. Patient selection was per the surgeon and patient decision, but generally included masses less than 7 cm in size. All locations, including hilar and posterior were included. Generally a four-arm approach was used with the da Vinci "s" system, although in selected cases, a 3 arm approach was used. A pure robotic approach was used in all cases, with no pure laparoscopic dissection. The surgical technique has been previously described. [12] Briefly, a medial camera port placement was used. The three arms utilized the robotic grasper, monopolar scissors, and a secondary grasper or atrial dual-blade retractor. The renal hilum was dissected and radiographic integration technology was used to identify the margins of resection. [13] The renal hilum was clamped with bulldog clamps under assistant control or with robotic control by the console surgeon; alternatively, the fourth robotic arm was used to clamp with an atraumatic robotic grasper.

The renal tumor was excised with shears and collecting system was oversewn with 2-0 vicryl (Ethicon, Cincinnati, USA) suture. The renal parenchyma was sutured with 0-vicryl or 1-vicryl suture. The method of renorraphy differed. In the first 13 patients, the assistant controlled the tension of renorraphy by placing a lapra-ty clip (Ethicon, Cincinnati, USA) on the renal parenchyma to cinch the suture tightly. This method is a duplication of the laparoscopic approach. The second method (patients 13–35) to perform renorrhaphy was with direct surgeon control by placing a 10 mm locking clip on the suture, and using the robotic needle driver to slide the clip down the suture to a desired tension under visual cues by the console surgeon. [14]

Postoperative management was routine. Patients were given narcotic medicine if they desired and ketorolac in selected cases. Low molecular weight heparin was given preoperatively after the first 6 cases for routine deep vein thrombosis prophylaxis. Patients were discharged home when tolerating a regular diet and when bowel function returned.

## Results

### Overall results

A total of 35 patients were identified. Clinical, pathological, and perioperative results are documented in table 1. Patients had a mean age of 62 and a mean tumor size of 2.8 cm on preoperative imaging. The operations were notable for mean warm ischemia time of 20 minutes and the renal collecting system was entered in 60% of cases. Final pathology revealed renal cell carcinoma in 66% of cases. All deep parenchymal margins were clear for cancer, and complications occurred in 4 patients. Additionally, there were two conversions to other procedures (as described below), which are not included in the series. There were no intraoperative complications.

**Table 1:** Demographic, operative, and pathological information from patients undergoing robotic assisted partial nephrectomy.

	Entire Series
Number	35
Mean Age (years)	62 (41–83)
Mean ASA Score	2.3 (1–3)
Mean BMI (kg/m2)	30.3 (21–38)
Mean Radiographic Tumor Size (cm)	2.8 (1–6)
Pelvicaliceal Repair	60%
Mean OR Time (min)	142 (69–219)
Mean Warm Ischemic Time (min)	21 (0–40)
Mean EBL (cc)	133 (25–500)
Mean Length of Stay (days)	2.5 (1–7)
Mean Pathological Size (cm)	2.5 (1–5)
Pathology: Renal Cell Cancer	66%
Pathology: Oncocytoma	6%
Pathology: Angiomyolipoma	14%
Pathology: Other Benign	14%
Margin	All negative
Elective Conversions	5%
Urgent/Emergent Conversions	0%

Complications	11%

### Conversions

One patient was converted from RPN to open partial nephrectomy on an elective basis when the margins were not clear during intraoperative ultrasound. This conversion occurred prior to clamping, but after hilar dissection. Open partial nephrectomy was completed without difficulty, with negative margins. Another patient was converted to robotic assisted cryoablation when, during the dissection, abundant adherent perinephric fat measuring 7 cm in depth could not be removed adequately, so complete ablation was performed without complication.

### Complications

Complications of RPN included one deep venous thrombosis, one myocardial infarction, two transfusions (1 for hematoma and 1 for medical reasons), and one patient with readmission for hypertensive crisis which was managed medically with re-institution of antihypertensive medicines.

The one patient with DVT/PE had undergone a 4-arm approach to robotic partial nephrectomy. He had not been given low molecular weight heparin prior to the operation, and his OR time was 190 minutes. After this patient (#6 in the series), low molecular weight heparin was routinely given with the operation.

The one myocardial infarction occurred in a patient who was cleared medically by her internist and cardiologist, but had a small asymptomatic MI which was detected with routine postoperative check of troponin levels. No intervention was needed.

One transfusion was for medical reasons. A second patient developed a clinical hematoma with a hematocrit of 26, and was transfused electively with no sequelae.

The last complication, a patient admitted for hypertensive crisis, had a long history of poorly controlled hypertension, and two weeks postoperatively was readmitted to control her pressure which had climbed above 200 mmHg.

## Discussion

Robotic partial nephrectomy, as an alternative to laparoscopic or open partial nephrectomy is still in a fledgling state with few reports and a technique which is not well choreographed. In this series, operative outcomes appear to be excellent, with comparable results to major laparoscopic series and previous small robotic series. [4,6-11]

Laparoscopic partial nephrectomy has been performed for > 10 years, but still has not gained wide popularity outside of major centers of excellence. The relative rarity of kidney cancer, combined with the advanced technical skills needed to reconstruct the kidney via laparoscopic means, likely have contributed to the challenges in popularizing minimally invasive nephron sparing surgery. Robotic partial nephrectomy has the advantage of making reconstruction of the kidney easier, and thus possibly more available to practicing urologists. Indeed, this is similar to the situation with robotic prostatectomy, which in a short time has gained immense popularity.

Disadvantages of RPN mirror those of LPN as well. The scepter of warm ischemia is ever present, though the "window" of safety is open to debate in the literature. [15-18]

In this series, warm ischemia was not a distinct disadvantage, as the mean warm ischemic time was low overall.

An additional theoretical disadvantage of RPN is the need for an assistant who is capable of comfortably placing instruments near the renal hilum. We found this last issue to be easily overcome, and routinely perform the procedure with residents and assistants of all levels.

Overall, RPN seems to have reasonable results for the treatment of small renal cell carcinomas, but randomized studies comparing it to the pure laparoscopic and open approaches are needed, and long term cancer control outcomes are needed. Cost issues regarding the expense of robotic assistance should also be addressed. [19]

## Conclusion

Robotic partial nephrectomy is still an operation in its infancy, but may build on the laparoscopic approach by offering ease of reconstruction after tumor excision. Wristed instruments and integration of imaging can aid in tumor dissection and excision. Preliminary surgical outcomes are reasonable, and long term oncological outcomes are pending.

## Competing interests

Dr. Bhayani has been a consultant for Intuitive Surgical and has been a consultant for Ethicon. There was no financing of this manuscript.

## Authors' contributions

SB performed the operations and wrote the manuscript. ND assisted with data collection.

## Pre-publication history

The pre-publication history for this paper can be accessed here:


